# Identification and characterization of *LIM* gene family in *Brassica rapa*

**DOI:** 10.1186/1471-2164-15-641

**Published:** 2014-08-03

**Authors:** Jong-In Park, Nasar Uddin Ahmed, Hee-Jeong Jung, Senthil Kumar Thamil Arasan, Mi-Young Chung, Yong-Gu Cho, Masao Watanabe, Ill-Sup Nou

**Affiliations:** Department of Horticulture, Sunchon National University, 255 Jungangno, Suncheon, Jeonnam 540-950 Republic of Korea; Department of Agricultural Education, Sunchon National University, 413 Jungangno, Suncheon, Jeonnam 540-742 Republic of Korea; Department of Crop Science, Chungbuk National University, 410 Seongbongro, Heungdokgu, Cheongju 361-763 Republic of Korea; Laboratory of Plant Reproductive Genetics, Graduate School of Life Sciences, Tohoku University, 2-1-1, Katahira, Aoba-ku, Sendai 980-8577 Japan

**Keywords:** *Brassica rapa*, Characterization, Expression analysis, *LIM* gene, Stress resistance

## Abstract

**Background:**

*LIM* (Lin-11, Isl-1 and Mec-3 domains) genes have been reported to trigger the formation of actin bundles, a major higher-order cytoskeletal assembly, in higher plants; however, the stress resistance related functions of these genes are still not well known. In this study, we collected 22 *LIM* genes designated as *Brassica rapa LIM* (*BrLIM*) from the *Brassica* database, analyzed the sequences, compared them with *LIM* genes of other plants and analyzed their expression after applying biotic and abiotic stresses in Chinese cabbage.

**Results:**

Upon sequence analysis these genes were confirmed as *LIM* genes and found to have a high degree of homology with *LIM* genes of other species. These genes showed distinct clusters when compared to other recognized LIM proteins upon phylogenetic analysis. Additionally, organ specific expression of these genes was observed in Chinese cabbage plants, with *BrPLIM2a, b, c, BrDAR1, BrPLIM2e, f* and *g* only being expressed in flower buds. Furthermore, the expression of these genes (except for *BrDAR1* and *BrPLIM2e*) was high in the early flowering stages. The remaining genes were expressed in almost all organs tested. All *BrDAR* genes showed higher expression in flower buds compared to other organs. These organ specific expressions were clearly correlated with the phylogenetic grouping. In addition, *BrWLIM2c* and *BrDAR4* responded to *Fusarium oxysporum* f. sp. *conglutinans* infection, while commonly two *BrDARs* and eight *BrLIMs* responded to cold, ABA and pH (pH5, pH7 and pH9) stress treatments in Chinese cabbage plants.

**Conclusion:**

Taken together, the results of this study indicate that *BrLIM* and *BrDAR* genes may be involved in resistance against biotic and abiotic stresses in *Brassica.*

**Electronic supplementary material:**

The online version of this article (doi:10.1186/1471-2164-15-641) contains supplementary material, which is available to authorized users.

## Background

LIM proteins have mainly been described in animals in which they play key roles in a variety of important developmental processes [1, 2]. These proteins contain one or several (up to five) double zinc finger motifs, known as LIM domains, which function by mediating protein-protein interactions [3, 4]. The three transcription factors, LIN11 from *Caenorhabditis elegans*[5], ISL-1 from rat [6] and MEC-3 from *C. elegans*[7], from which the acronym LIM is derived, operate during transcription in association with a DNA-binding homeodomain. LIM domains have since been found in a wide variety of eukaryotic proteins of diverse functions. LIM domain-containing transcription factors without the homeodomain have also been described. Specifically, the LIM-only protein LMO2 was found to act as a bridging molecule in assembly of the erythroid DNA-binding complex [8], while other LIM-domain-containing proteins such as LIM kinases are known to participate in regulation of actin dynamics through phosphorylation of cofilin [9, 10]. In contrast, plants possess two distinct sets of LIM proteins, one that is plant–specific and has been partially functional characterized [11, 12]. Another, cysteine rich protein (CRP)-like that comprises CRPs exhibiting the same overall structure found in animals (i.e., two very similar LIM domains separated by an ≈ 50 amino acid–long interLIM domain and a relatively short and variable C–terminal domain) [13].

Plants have evolved mechanisms to exploit, survive, or minimize the negative impact of a diverse range of environmental factors including potential pathogens, and in many cases the plant cytoskeleton is instrumental in mediating the plant’s response. Changes in organization of the plant cytoskeleton during plant interactions with these factors are complex and varied, and much still remains to be elucidated, especially in terms of the molecules that signal and induce the dramatic reorganizations that are often observed. Physical and chemical barriers resulting from actin-dependent cytoplasmic aggregation, secretion, and papilla formation appear to constitute an important and probably ancient form of basal resistance to pathogen attack [14]. Reorganization of cytoskeletal elements may also be a mechanism responsible for abiotic stress responses. For example, cytoskeletal elements translocate chloroplasts under high light conditions [15], facilitate gravity sensing [16], and direct cellular response to wounding [17, 18]. A full understanding of the cytoskeletal basis of cytoplasmic aggregation will require identification and characterization of proteins involved in the signaling pathways those induce cytoskeletal rearrangement and responsible proteins for bringing about cytoskeletal reorganization and their function. This mechanism utilizes regulatory proteins that generate intracellular motility, such as myosin [19] and thick microfilament bundles focusing on the site of infection, such as the actin-bundling proteins villin and fimbrin [20, 21].

In the cytoplasm, actin monomers polymerize into actin filaments (AFs), which constitute the core elements of the actin cytoskeleton, providing mechanical support to the cytoplasm and serving as tracks for myosin-dependent intracellular transport [22, 23]. To fulfill its various roles, the actin cytoskeleton requires a sophisticated regulatory system to control its organization and dynamics at both spatial and temporal levels. Primary components of this system are the actin binding proteins (ABPs) that directly interact with monomeric and/or polymerized actin to promote AF nucleation, polymerization, depolymerization, stabilization, severing, capping, and cross-linking [24]. Recently, a number of vertebrate LIM domain proteins belonging to the Cys-rich protein (CRP) family and several structurally related plant proteins (hereafter referred to as plant LIMs) have been shown to function as ABPs [25–28]. In *Arabidopsis*, two distinct *LIM* gene subfamilies have been reported. The first subfamily contains six genes, which encode proteins with two LIM domains that share similarity with animal CRPs [29]. These LIM domain-containing proteins bind actin filaments and influence actin cytoskeleton organization [29]. *In vitro*, chicken CRP1 and tobacco (*Nicotiana tabacum*) WLIM1 bind directly to AFs to trigger the formation of thick actin bundles. Importantly, over-expression of CRP1 and WLIM1 proteins has been shown to be sufficient to significantly increase the bundling of AFs in rat fibroblasts and tobacco cells, respectively [13, 26, 27]. The genes in the second subfamily encode DA1 and DA1 related (DAR) proteins with a single LIM domain and are specific to plants [11]. DA1 encodes a putative ubiquitin receptor with two ubiquitin interacting motifs (UIMs) and a single LIM domain, while e.g. DAR2 contains a single LIM domain and a putative zinc-binding domain but lacks UIMs suggesting a possible functional divergence with DA1 [30]. The members of this family are involved in diverse functions e.g. seed and organ size control, root meristem size control, resistance signaling and cold response [11, 30, 31].

In this study, we retrieved 22 *Brassica rapa LIM* genes from the *Brassica* database, analyzed the sequences and studied their homology with *LIM* genes of other species. We also analyzed their phylogenetic classification and discussed their correlation with organ specific expression. Expression of these genes was also analyzed following application of different biotic and abiotic stresses and their association was discussed with biotic and abiotic stress resistance in *Brassica*.

## Results and discussion

### Sequence analysis and phylogenetic classification

We identified 22 LIM proteins, designated as *B. rapa* LIM (BrLIM), after which the sequences were analyzed (Table 1). The predicted size of the 22 BrLIMs ranged from 92 to 1315 amino acids (10.36 to 150.61 kDa), and the predicted isoelectric points varied from 5.28 to 9.77. Analysis of the protein domain organization showed that proteins contained the characteristic LIM domain in the conserved region. Additionally, genomic DNA sequence of the 22 *BrLIM* genes was determined from the *B. rapa* chromosome sequences and the introns and exons were identified by sequence analysis. Structural information pertaining to the 22 *BrLIM* genes is presented in Additional file 1: Figure S1. This analysis confirmed that the identified genes code for LIM domain containing proteins. A BLAST search of the NCBI database conducted for comparison of these *BrLIM*s with *LIM* genes of other species revealed that the deduced amino acid sequences of 22 BrLIMs shared high homology, primarily with LIMs of *A. thaliana* and some other homologous species (Table 2). Pairwise amino acid sequence comparisons among these 22 BrLIM proteins were also calculated using BLAST of the NCBI database and shown in Additional file 2: Table S2. The similarity within these BrLIM protein sequences ranged from 23 to 99% and 8 BrLIMs showed above 90% similarity within the species indicating their probable duplication.

**Table 1:** ***In silico*** **analysis of** ***LIM*** **genes collected from the** ***Brassica*** **database** ^**a**^

Name of genes	Accession number	ORF (bp)	Located chromosome number	Protein			
				Length (aa)	LIM domain Start - end (aa)	Mol.Wt. (KD)	pI
*BrLIM1*	Bra014447	636	A04	211	9-61 and 105-157	23.62	6.2
*BrLIM2*	Bra007586	654	A09	217	9-61 and 104-156	24.24	5.83
*BrLIM3*	Bra033233	618	A10	205	9-61 and 103-155	22.87	7.12
*BrLIM4*	Bra032650	618	A09	205	9-61 and 103-155	22.92	6.77
*BrLIM5*	Bra007189	606	A09	201	10-62 and 109-161	21.88	9.09
*BrLIM6*	Bra026940	279	A09	92	10-62	10.36	8.93
*BrLIM7*	KJ686594^b^	603	A04	200	10-62 and 108-160	21.88	9.16
	Bra014721^c^						
*BrLIM8*	Bra005003	600	A05	199	9-61 and 108-160	21.83	8.92
*BrLIM9*	KJ686594^b^	603	A03	200	9-61 and 108-160	21.92	8.92
	Bra000154^c^						
*BrLIM10*	Bra019956	573	A06	190	9-61 and 109-161	21.06	9.11
*BrLIM11*	Bra018454	570	A05	189	9-61 and 109-161	20.98	9.02
*BrLIM12*	Bra037160	1173	A09	390	38-93	44.26	5.68
*BrLIM13*	Bra037159	1551	A09	516	147-209	59.32	6.10
*BrLIM14*	Bra016514	1389	A08	462	102-154	53.23	6.34
*BrLIM15* ^*d*^	Bra025725	1584	A06	527	166-218	59.68	5.89
*BrLIM16* ^*d*^	Bra011712	1797	A01	598	230-282	68.09	5.30
*BrLIM17*	Bra005011	1506	A05	501	160-224	56.31	8.19
*BrLIM18*	Bra000152	1545	A03	514	146-198	57.99	7.30
*BrLIM19* ^*e*^	Bra012087	3948	A07	1315	471-526	150.61	5.28
*BrLIM20*	Bra000404	681	A03	226	9-61 and 103-155	24.93	6.31
*BrLIM21*	Bra039313	690	A04	229	9-61 and 105-157	25.13	6.04
*BrLIM22*	Bra004939	690	A05	229	9-61 and 105-157	25.03	6.12

^a^ *Brassica* database (http://brassicadb.org/brad/index.php), ^b^Genbank accession number, ^c^ *Brassica* database accession number, ^d^Additional 2 UIM domain and ^e^Additional 4 UIM domain.

**Table 2:** **Homology analysis of** ***LIM*** **and DA1-related (** ***DAR*** **) genes of** ***Brassica rapa*** ^**a**^

Name of genes	New given name	Top matched LIM clones	Name of proteins	Identity (%)	E value	Homologous species	References
*BrLIM1*	*BrPLIM2a*	NP182104	PLIM2a	77	6e-113	*Arabidopsis thaliana*	[32]
		ACB05475	LIM protein 2b	68	1e-105	*Nicotiana tabacum*	[33]
*BrLIM2*	BrPLIM2b	NP182104	PLIM2a	87	2e-112	*A. thaliana*	[32]
		ABK58466	PLIM2a	80	6e-106	*Populus tremula x Populus alba*	Unpublished
*BrLIM3*	BrPLIM2c	XP002892043	LIM protein	95	7e-144	*Arabidopsis lyrata subsp. lyrata*	Unpublished
		ABK58466	PLIM2a	74	2e-99	*P. tremula x Populus alba*	Unpublished
*BrLIM4*	BrPLIM2d	NP182104	PLIM2a	72	1e-99	*A. thaliana*	[32]
		XP002892043	LIM protein	92	2e-143	*A. lyrata subsp. lyrata*	Unpublished
*BrLIM5*	*BrWLIM2a*	XP002879831	LIM protein	88	2e-119	*A. lyrata subsp. lyrata*	Unpublished
		WLIM2a	LIM protein	87	4e-118	*A. thaliana*	[32]
*BrLIM6*	*BrWLIM2b*	XP002876333	LIM protein	91	8e-52	*A. lyrata subsp. lyrata*	Unpublished
		AAD56951	WLIM2	83	3e-47	*N. tabacum*	Unpublished
*BrLIM7*	*BrWLIM2c*	XP002876333	LIM protein	93	3e-122	*A. lyrata subsp. lyrata*	Unpublished
		ABK58469	WLIM2a	84	1e-111	*P. tremula x Populus alba*	Unpublished
*BrLIM8*	*BrWLIM2d*	NP181519	WLIM2a	93	6e-124	*A. thaliana*	[32]
		ACX47456	LIM1	84	2e-113	*Hevea brasiliensis*	Unpublished
*BrLIM9*	*BrWLIM2e*	XP002879831	LIM protein	91	1e-125	*A. lyrata subsp. lyrata*	Unpublished
		ABK58469	WLIM2a	81	4e-114	*P. tremula x Populus alba*	Unpublished
*BrLIM10*	*BrWLIM1a*	NP172491	WLIM1	99	1e-137	*A. thaliana*	[34]
		ABB51614	LIM protein	97	6e-133	*Brassica napus*	Unpublished
*BrLIM11*	*BrWLIM1b*	ABB51614	LIM protein	99	6e-137	*B. napus*	Unpublished
		NP172491	WLIM1	98	6e-134	*A. thaliana*	[34]
*BrLIM12*	*BrDAR1*	AED98240	DAR6 protein	67	0.0	*A. thaliana*	[35]
		AED98241	DAR5 protein	64	5e-160	*A. thaliana*	[35]
*BrLIM13*	*BrDAR2*	AED98243	DAR3 protein	65	0.0	*A. thaliana*	[35]
		XP003611877	LIM and UIM domain-containing	43	2e-106	*Medicago truncatula*	Unpublished
*BrLIM14*	*BrDAR3*	NP173361	DA1 protein	71	0.0	*A. thaliana*	[34]
		EOY14670	LIM and UIM domain-containing	65	0.0	*Theobroma cacao*	Unpublished
*BrLIM15*	*BrDAR4*	NP173361	DA1 protein	87	0.0	*A. thaliana*	[34]
		EOY14670	LIM and UIM domain-containing	66	0.0	*T. cacao*	Unpublished
*BrLIM16*	*BrDAR5*	NP195404	DAR1	89	0.0	*A. thaliana*	[36]
		XP003611877	LIM and UIM domain-containing	64	0.0	*M. truncatula*	Unpublished
*BrLIM17*	*BrDAR6*	NP181513	DAR2	86	0.0	*A. thaliana*	[32]
		XP003616507	LIM and UIM domain-containing	61	0.0	*M. truncatula*	Unpublished
*BrLIM18*	*BrDAR7*	NP181513	DAR2	89	0.0	*A. thaliana*	[32]
		XP003616507	LIM and UIM domain-containing	66	0.0	*M. truncatula*	Unpublished
*BrLIM19*	*BrDAR8*	AED98240	DAR6 protein	57	1e-136	*A. thaliana*	[35]
		NP195404	DAR1	42	2e-51	*A. thaliana*	[36]
*BrLIM20*	*BrPLIM2e*	NP182104	PLIM2a	83	9e-121	*A. thaliana*	[32]
		AAF75828	PLIM2	70	2e-84	*N. tabacum*	Unpublished
*BrLIM21*	*BrPLIM2f*	XP002882034	LIM protein	86	1e-141	*A. lyrata subsp. lyrata*	Unpublished
		NP182104	PLIM2a	86	5e-132	*A. thaliana*	[32]
*BrLIM22*	*BrPLIM2g*	NP182104	PLIM2a	88	6e-133	*A. thaliana*	[32]
		ABK58466	PLIM2a	77	2e-99	*P. tremula x Populus alba*	Unpublished

^a^Analyzed using BLAST from NCBI, http://www.ncbi.nlm.nih.gov/BLAST/.

**Figure 1:**
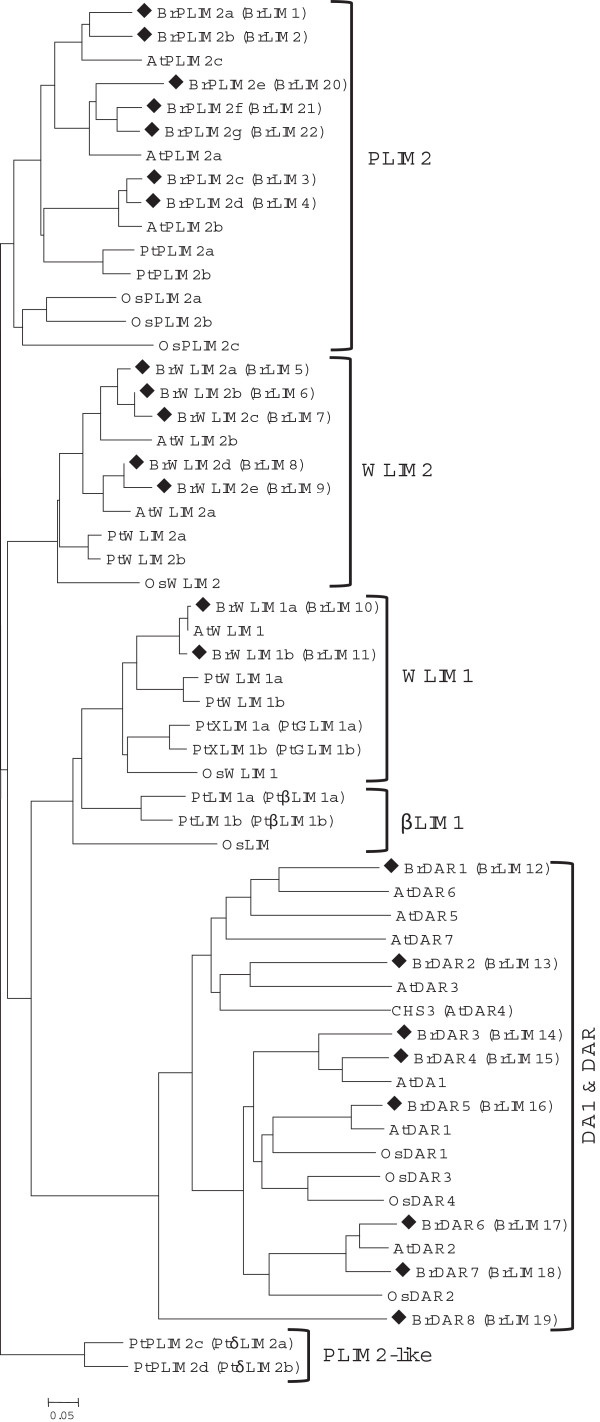
**Phylogenetic tree showing the relatedness of the deduced full-length amino acid sequences of 22 BrLIM proteins and all LIM family proteins of** ***A. thaliana*** **,** ***Populus trichocarpa*** **and** ***Oryza sativa*** **.** BrLIM proteins are indicated by square bullets. The scale represents the frequency of amino acid substitutions between sequences as determined by the Poisson evolutionary distance method. A species acronym is added before each LIM protein name: At, *A. thaliana*; Pt, *Populus trichocarpa* and Os, *Oryza sativa*. The old nomenclature of LIM genes are indicated under bracket. Details of each gene are given in Additional file 3: Table S3.

**Figure 2:**
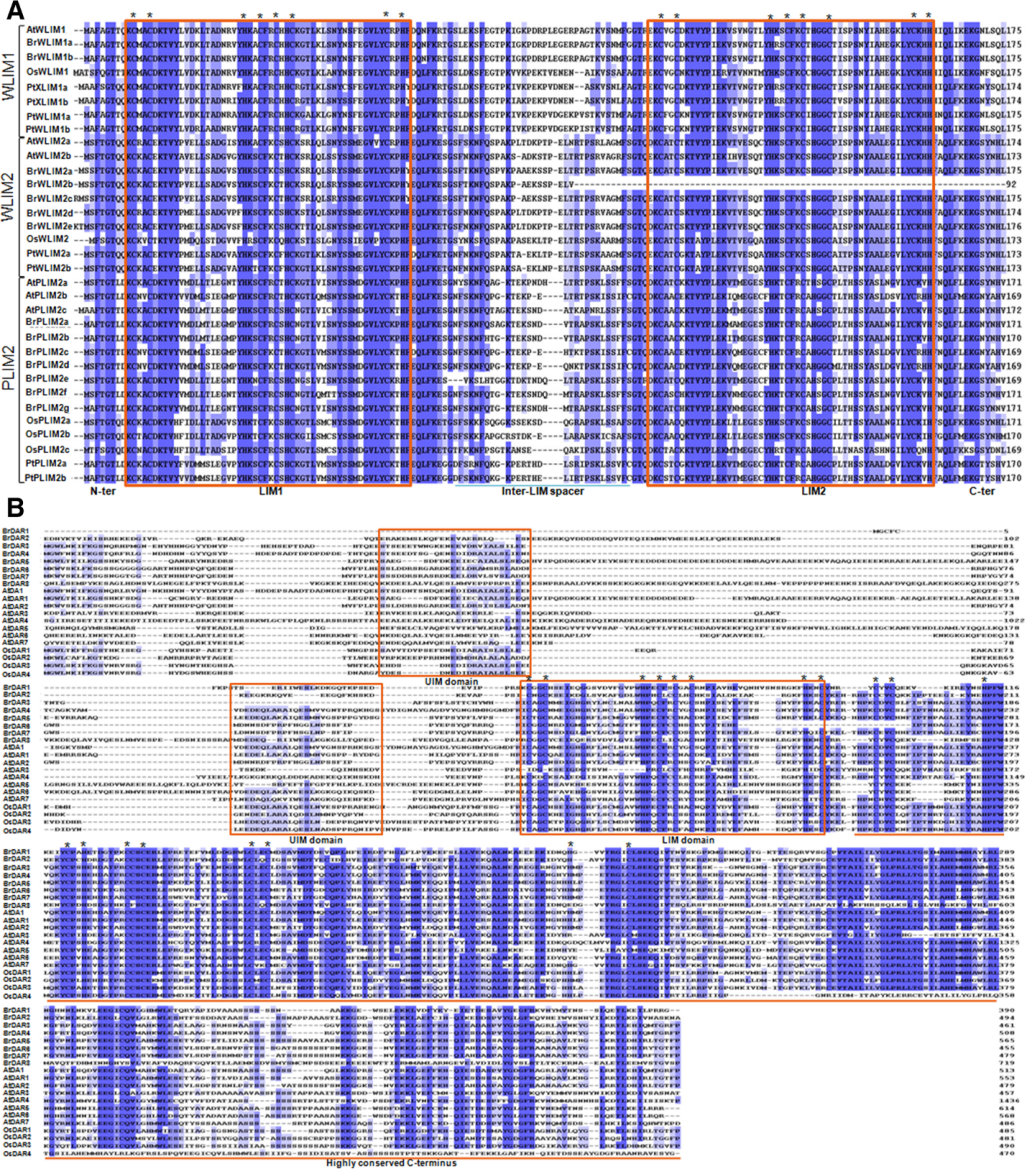
**Alignment of deduced amino acid sequences of A) LIM and B) DAR protein groups using PIR.** Solid boxes indicate UIM and LIM domains. Underline indicates highly conserved C-terminus. Asterisks show conserved cysteine and histidine residues (zinc-binding motifs) in the highly conserved LIM domains and C-terminus. Numbers in the right margin indicate the positions of amino acid residues. Identical amino acids are indicated by a dark background, while > 50% similarities are indicated by a light background.

We also retrieved all LIM family protein sequences of *A. thaliana, Populus trichocarpa* and *Oryza sativa* from NCBI and constructed a phylogenetic tree with 22 deduced amino acid sequences of BrLIM using the NJ method (Figure 1, Additional file 3: Table S3). In the phylogenetic tree, previously identified LIM1 and LIM2 groups [37] were clearly separated and the plant LIM family was further divided into four groups, αLIM1, βLIM1, γLIM2, and δLIM2, resulting from division of the LIM1 and LIM2 groups. This phylogenetic analysis confirms the existence of the PLIM1, WLIM1, PLIM2, and WLIM2 subgroups as previously described [37]. The αLIM1 group includes the PLIM1 and WLIM1 subgroups, while βLIM1 is a newly identified group [38]. The WLIM2 and PLIM2 subgroups belong to the γLIM2 and δLIM2 groups, respectively. Among the 22 BrLIMs, BrLIM10 and 11 were clustered with WLIM1 subgroup under the αLIM1 group. BrLIM5, 6, 7, 8 and 9 were separated with the WLIM2 subgroup under the γLIM2 group. Moreover, BrLIM1, 2, 3, 4, 20, 21 and 22 separated with the PLIM2 subgroup under the δLIM2 group, showing close relationships with the LIM sequences of other species. Multiple alignments among members of the WLIM1, WLIM2, PLIM2 and DAR groups also showed clearly distinct amino acid residues conserved in the domains and other regions of their protein sequences (Figure 2). In *Arabidopsis,* LIM proteins are encoded by genes from two distinct subfamilies, one encoding proteins with two LIM domains that are homologous to animal CRP proteins [37]. Consequently, highly homologous WLIM1, PLIM2, and WLIM2 proteins of *B. rapa* of this subfamily also contain two LIM domains except BrLIM6. Interestingly, with two LIM domains N-terminal extensions were observed in BrLIM7 and 9 protein sequences and BrLIM9 had additional 11 transmembrane domains in the N-terminal extension (Figure 2, Table 1). These N-terminal extensions suggest probable misannotation or divergent functions of these two genes which require further experimental evidence.

To investigate the existence of N-terminal extensions of *BrLIM7* and *9*, we analyzed expression of both genes over different organs specifying forward and reverse primers at several locations of the sequences (Additional file 1: Figure S2). Both genes showed expression, when forward (#5 of *BrLIM7* and #7 of *BrLIM9*) and reverse (#6 of *BrLIM7* and #8 of *BrLIM9*) primers were selected at the conserved parts. Conversely, no expression was found, when the forward primers (#1 of *BrLIM7* and #3 of *BrLIM9*) were selected at the extended parts and the reverse primers (#3 and #4 of *BrLIM7*; #5 and #6 *BrLIM9*) at several locations of the conserved parts. Subsequently, 3’-RACE PCR using primer #1 of *BrLIM7* and primer #3 of *BrLIM9* also showed discontinuation of the transcription towards conserved parts and found to show polyA tails indicating the end of the transcript. Alongside, genomic DNA amplification specifying these transition portions of both genes revealed no sequence distortion. Thus, it is clear that the extended portions are not part of *BrLIM7* and *9*. It was a wrong annotation of the splice sites in the original prediction. The correct exons of *BrLIM7* and *9* were then drawn in the postulated gene structures (Additional file 1: Figure S2) and the primer combinations, #5 and #6 of *BrLIM7* and #7 and #8 of *BrLIM9* located at the corrected exons, were used in latter expression studies. These corrected sequences were deposited in the genbank under the accession numbers, KJ686594 (*BrLIM7*) and KJ686595 (*BrLIM9*). These corrected sequences are the basis for all further analysis and all figures and tables were corrected accordingly.

The genes in the second subfamily encode DA1 and DA1-related (DAR) proteins specific to plants contain a single conserved LIM domain [11]. Interestingly, BrLIM12, 13, 14, 15, 16, 17, 18 and 19 were clustered into a new group, showing close relationships with eight DA1 and DAR sequences of *A. thaliana* and four DAR sequences of *O. sativa*. Additionally, all members of this new group contain only one LIM domain, while some also contain one or more UIM(s). Therefore, BrLIM proteins were renamed according to their phylogenetic relationship as *B. rapa* DAR (BrDAR) (Table 2). Alignment of the motif sequences of the DA1 and DAR group showed very low similarity with WLIM1, WLIM2 and PLIM2 sequences, indicating their distinctness from these groups (Figure 2). However, the BrDARs were highly similar to DA1 and DAR sequences of *A. thaliana* and *O. sativa*, and these all contained similar patterns of motifs in their conserved regions. DA1 of *A. thaliana* contains two UIMs, a single LIM domain and the highly conserved C-terminal region [11]. In contrast, DAR2 contains a single LIM domain and a putative zinc-binding domain but lacks UIMs compared with DA1 and DAR1 [30]. Similarly, BrDAR4 and 5 contain two UIMs and a single LIM domain, while BrDAR8 contains four UIM and a single LIM domain with highly conserved C-terminal region in their protein sequences. Other BrDARs contain only a single LIM domain and highly conserved C-terminal regions (Table 1). Thus, the *BrLIM* genes showed high similarity with corresponding groups of *LIM* genes of different species, indicating their close evolutionary relationship.

### Organ specific expression analysis under unstressed condition

**Figure 3:**
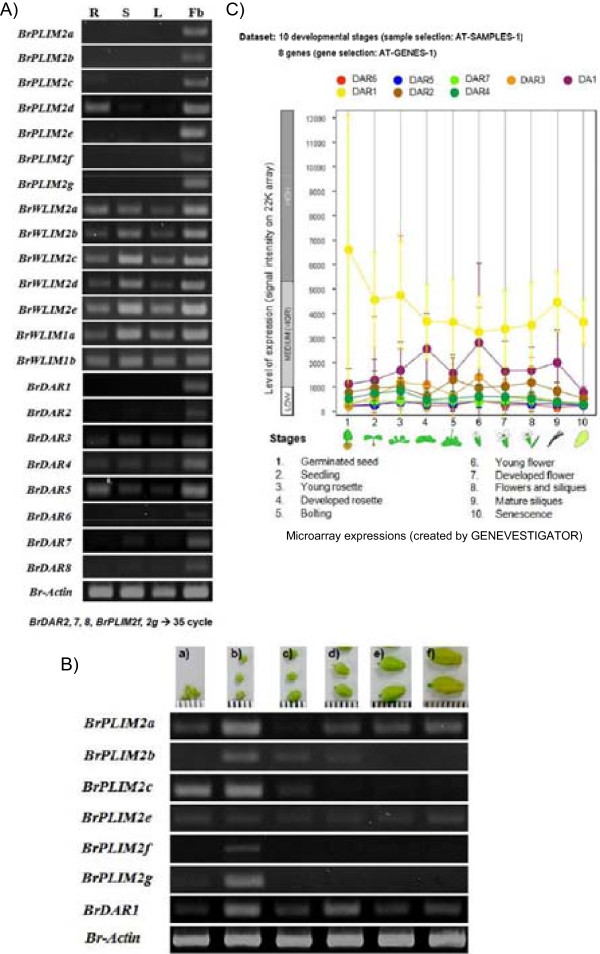
**Expression analysis of 14** ***BrLIM*** **and 8** ***BrDAR*** **genes, A) over different organs, B) at six growth stages (a-f) of flower buds of** ***Brassica rapa*** **‘SUN-3061’ plants and C) microarray expressions of** ***DA1*** **and seven** ***DAR*** **genes of** ***Arabidopsis thaliana*** **at 10 developmental stages.** Lanes 1–4, PCR products of roots (R), stems (S), leaves (L) and flower buds (Fb). a-f, young to mature stages of flower buds.

Expression analysis was performed using specific primers with equal amounts of cDNA templates prepared from the mRNA of roots, stems, leaves and flower buds of healthy Chinese cabbage plants without applying any stresses by RT-PCR and organ specific expression was observed. Specifically, *BrPLIM2d, BrWLIM2a, b, c, d, e, BrWLIM1a* and *b* showed expression in all the organs tested. *BrPLIM2a, b, c, BrPLIM2e, f* and *g* were only expressed in flower buds (Figure 3A). Expression of the genes that were only expressed in flower buds was further analyzed during six developmental stages (from the very young bud stage to the mature bud stage), and all genes except *BrPLIM2e* showed higher expression in young buds (Figure 3B). This expression pattern and phylogenetic classification revealed two clear groups of *BrLIM* genes, with one group (*BrPLIM*s except *BrPLIM2d*) only being expressed in flower buds and another group (WLIM) being expressed in all vegetative parts of the Chinese cabbage plants tested in this study. Thus, organ specific expressions of BrLIMs showed clear correlations with phylogenetic grouping as well.

DAR subfamily genes of *B. rapa* showed a different expression pattern compared to other LIM domain containing genes. Specifically, *BrDAR3, 4* and *5* showed expression in all the organs tested. *BrDAR2, 7* and *8* showed expression in all organs except roots, while *BrDAR1* was only expressed in flower buds. Noticeably, all *BrDAR* genes expressed highly in flower buds compared to other organs (Figure 3A). Expression of *BrDAR1* that was only expressed in flower buds was further analyzed during six developmental stages (from the very young bud stage to the mature bud stage), and found higher expressions in young and middle aged buds (Figure 3B). Microarray expression of *DA1* and *DAR* genes of *A. thaliana* were also lower at different vegetative parts compared to floral organs and comparatively higher at young flower bud stages (Figure 3C).

The PLIM-1 protein of sunflower appeared in the microspore stage in a limited number of cytoplasmic bodies, became undetectable in bicellular pollen, and reappeared in tricellular pollen grains in cortical patches, being particularly concentrated in the F-actin-enriched germination cones of the vegetative cell. Taken together, these findings suggest a dual function of *PLIM-1* gene during pollen development [39]. Conversely, expression of *NtPLIM1* of tobacco in germinating pollen suggests a possible role in pollen germination and/or pollen tube growth [40]. LiLIM1, an effective actin bundling protein, also plays an important role in pollen tube growth of *Lilium longiflorum*[28]. Likewise, the *BrPLIM*s expressed in young flower bud stage might play a role in the development of pollen tube. Earlier studies have also suggested the existence of two main *LIM* gene subfamilies that differ in their expression patterns [37]. Again, based on phylogenetic analysis of 149 LIMs and comparison of the available expression data, a complex classification of *LIM* genes has been proposed by Arnaud et al., [38]. According to this classification, the *Arabidopsis LIM* gene family comprises three vegetative (*WLIM1* and *WLIM2a* and *b*) and three reproductive (*PLIM2a-c*) isoforms, of which, all *PLIMs* have been shown to be expressed in pollen [41, 42]. Ye et al. [42] also suggested the critical roles of *Arabidopsis PLIMs* in actin configuration during pollen germination and tube growth. Specific control of PLIM actin regulatory activities by pH is particularly relevant with regard to the potential biological functions of these proteins in growing tip region of pollen tubes [29].

### Expression analysis under stress conditions

#### Biotic stress

**Figure 4:**
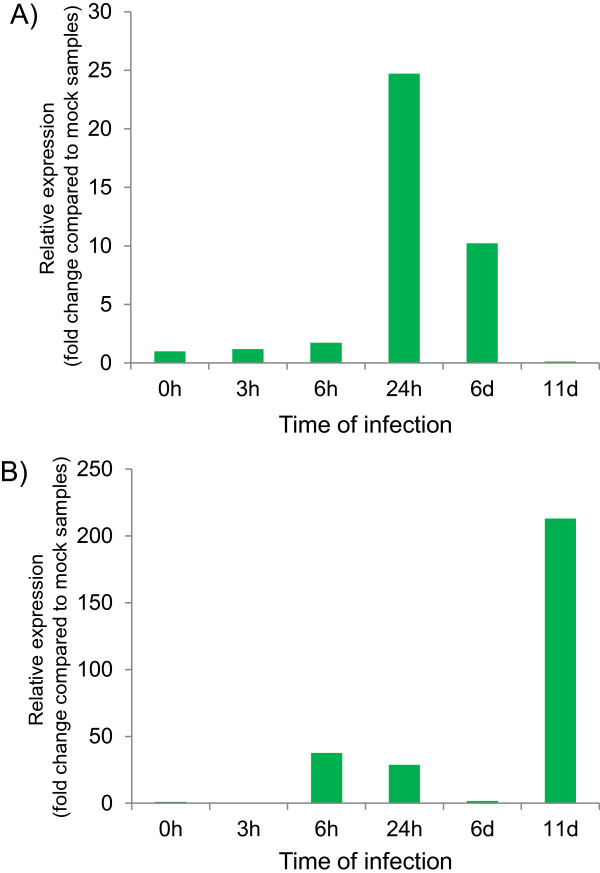
**Real-time quantitative PCR expression analysis of A)** ***BrWLIM2c*** **and B)** ***BrDAR4*** **after infection of Chinese cabbage (** ***Brassica rapa*** **‘SUN-3061’) with** ***Fusarium oxysporum*** **f. sp.** ***Conglutinans.*** Relative gene expressions in treated samples are presented as fold change compared to mock treated samples. At each time point gene expression values in mock treated samples are set to 1.

Biotic stress responses are a crucial issue for the *Brassica* crops and functions of *LIM* genes in biotic stress responses are not well studied yet. We investigated the responses of the 22 *BrLIM* genes identified in this study to infection by *F. oxysporum* f.sp. *conglutinans* in Chinese cabbage after various times. *BrWLIM2c* and *BrDAR4* were found to be up-regulated with the time course of infection. Specifically, *BrDAR4* showed very high responses in the late stages of fungal infection, while *BrWLIM2c* responded during earlier stages (Figure 4). *AtWLIM1, AtWLIM2b, AtDAR7, AtDAR4* and *AtDAR1* genes also showed responses during microarray expression analysis after infection with *F. oxysporum* in *A. thaliana*, which were collected from public database (Additional file 4: Figure S3). We also analyzed the expression of all *BrLIMs* after infection with the soft rot disease causing necrotroph bacteria, *Pectobacterium carotovorum* subsp. *carotovorum*; however, no differential expression was observed in Chinese cabbage plants (data not shown). These results suggest probable involvement of *BrWLIM2c* and *BrDAR4* with *F. oxysporum* f.sp. *conglutinans* resistance and other *BrLIM* and *BrDAR* genes might also have association with resistance against other biotic stress agents in *Brassica* crops.

Plants encounter and must deal with a range of other organisms that may be potential partners or pathogens, and the plant cytoskeleton plays a key role in their response to such organisms. Cell wall appositions, or papillae, are important barriers formed by plants in defense against attempted penetration by fungal and oomycete pathogens [43]. Prior to the development of papillae, plant cytosol and subcellular components are rapidly translocated to the site of pathogen penetration via a mechanism known as cytoplasmic aggregation that is dependent on the action of the actin component of the cytoskeleton [44, 45]. This cytoplasmic aggregation has been observed in many plant-microbe interactions [46] and is a common resistance response to pathogens by both dicotyledonous and monocotyledonous plants to invading filamentous pathogens. Microfilaments are the main cytoskeletal element responsible for penetration resistance based on cytoplasmic aggregation and papilla formation at the site of infection. For example, focusing of actin microfilaments at the penetration site is more extensive in resistant plants than susceptible hosts in flax (*Linum usitatissimum*)-Melampsora and barley (*Hordeum vulgare*)-Erysiphe interactions [47, 48], suggesting that accumulation of material at the site of infection is related to the degree of host resistance. Some other previous observations suggest that inhibition of penetration through cytoplasmic aggregation and papilla formation is an early, if not the first, tactic in plant resistance, and that it may be backed up by the hypersensitive response [49]. Thus, the physical and chemical barrier resulting from actin-dependent cytoplasmic aggregation, secretion, and papilla formation appears to constitute an important and probably ancient form of basal resistance to pathogen attack [14]. For a full understanding of the cytoskeletal basis of cytoplasmic aggregation, it is very essential to elucidate the role of responsible regulatory proteins that participate in other forms of cytoplasmic streaming, as well as additional factors specific for the localized defense response. Proteins those generate intracellular motility (e.g., myosin) are likely to be important components of such regulations [19]. In addition, several CRP-like plant LIM proteins have been shown to function as ABPs [25–28], and *LiLIM1* has been proposed as an effective ABP, that plays an important role in pollen tube growth of *L. longiflorum*[28]. *In vitro*, tobacco (*N. tabacum*) WLIM1 binds directly to AFs and triggers the formation of thick actin bundles. Importantly, overexpression of WLIM1 proteins was sufficient to significantly increase the bundling of AFs in tobacco cells [13, 27]. Thus, actin binding and bundling function of *LIM* genes could also be an important factor for actin-dependent resistance mechanism against pathogen infection. In this study, high expression of *BrWLIM2c* gene against pathogen infection is tempting to speculate that BrLIM protein may also be implicated in the perception of pathogen resistance mechanism.

Genes in the second subfamily encode DA1 proteins, which are specific to plants. The Arabidopsis LIM domain protein DA1 was characterized to function as an ubiquitin receptor [11]. Ubiquitin is a highly conserved and wide-spread small protein modifier that is engaged in a wide range of cellular [50] and biological processes, such as resistance to disease and abiotic stresses [51–53]. The consequences of ubiquitination are accusing the target protein to proteolysis or relocalization or endocytosis [54]. DA1 is inevitably involved in ubiquitination resulting extension of the cellular proliferation period, thus increasing cell numbers and ultimately plant organ size [11, 12]. Our highly expressed *BrDAR4* in response to pathogen infection might be involved in biotic stress resistance of *B. rapa* following aforesaid mechanism. Another member of this family CHS3/AtDAR4 containing TIR-NBS-LRR domain is associated with resistance signaling and cold response [31] and AtDAR2 with controlling of root meristem size [30]. Alongside, no BrDAR protein contained TIR-NBS-LRR domain like as *CHS3/AtDAR4.* However, reports on the responses of *LIM* genes to biotic stresses are still lacking, and this is the first report of such possible role of LIM domain containing genes against biotic stress caused by *F. oxysporum* f.sp. *conglutinans* in Chinese cabbage. Thus, *BrLIM* and *BrDAR* genes might be involved in biotic stress resistance related functions in *B. rapa*.

#### Abiotic stresses

**Figure 5:**
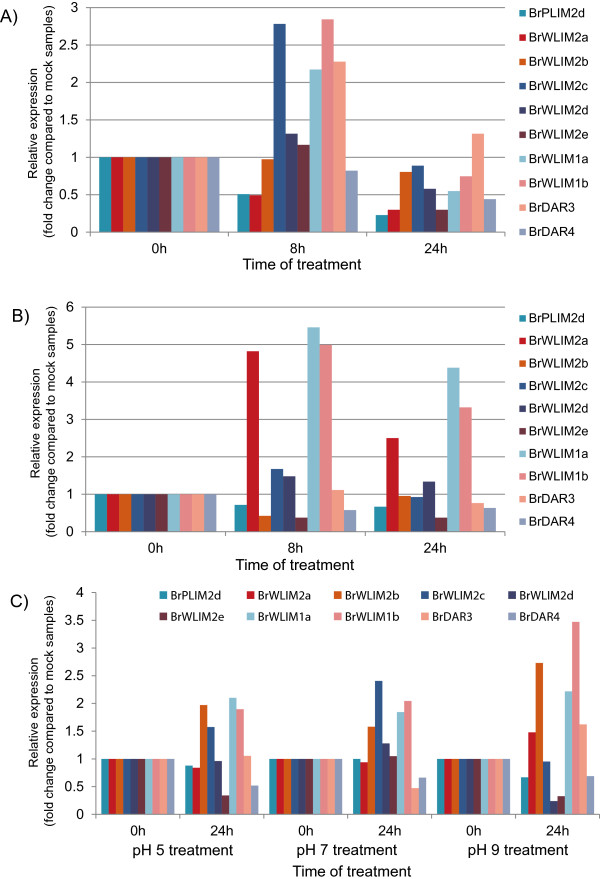
**Real-time quantitative PCR expression analysis of** ***BrLIM*** **and** ***BrDAR*** **genes after A) cold, B) ABA and C) pH stress treatments in Chinese cabbage (** ***Brassica rapa*** **‘SUN-3061’)** ***.*** Relative gene expressions in treated samples are presented as fold change compared to mock treated samples. At each time point gene expression values in mock treated samples are set to 1.

In addition to biotic stresses, expression of *BrLIMs* was also analyzed in response to abiotic stress conditions such as cold, ABA and pH stresses. Commonly ten *BrLIMs* among them, showed differential expressions in response to the abiotic stresses applied in this study. Specifically, *BrPLIM2d, BrWLIM2a, b, c, d, e, BrWLIM1a, b, BrDAR3* and *4* showed differential expression after cold, ABA and pH stress treatments compared to mock treated samples (control) of Chinese cabbage (Figure 5). *BrPLIM2d, BrWLIM2a, b* and *BrDAR4* were down-regulated in response to cold stress throughout the treatment period, while *BrWLIM2c, d, e, BrWLIM1a, b* and *BrDAR3* were up-regulated during the first 8 h of treatment, after which they were down-regulated. Following ABA stress treatments, *BrPLIM2d, BrWLIM2b, e* and *BrDAR4* were also down-regulated throughout the treatment period, while *BrWLIM2a, c, d, BrWLIM1a, b* and *BrDAR3* were up-regulated during the first 8 h of treatment, after which they were down-regulated. Moreover, *BrPLIM2d, BrWLIM2a* and *BrDAR4* were down-regulated and *BrWLIM1a, b BrWLIM2b* and c were up-regulated throughout the treatment period in response to pH 5, 7 and 9 stress treatments compared to mock treated samples (control) which were maintained after transferring into a fresh medium with pH 5.8. *BrWLIM2d* and *BrWLIM2e* were down-regulated in response to pH 5 and 9, while was up-regulated in response to pH 7. *BrDAR3* was down-regulated in response to pH 7 and up-regulated in response to pH 5 and 9. Interestingly, *BrPLIM2d* and *BrDAR4* were down-regulated and *BrWLIM1a, b* and *BrWLIM2c* were up-regulated in response to all abiotic stress treatments applied in this study. *AtWLIM1, AtWLIM2a, AtWLIM2b, DAR7, DAR4, DAR2* and *DAR1* genes also showed responses after cold stress treatments in *A. thaliana* during microarray expressions study collected from public database (Additional file 4: Figure S4).

Plants actin cytoskeleton is a major signaling target and in response to numerous abiotic and biotic stimuli, it changes dramatically in various ways, ranging from filament bundling, to massive actin depolymerization, to assembly of new filament arrays [55–57]. Plant cells can detect mechanical stress on the epidermal cell surface and respond by reorganizing subcellular components like, actin microfilaments, endoplasmic reticulum (ER) and peroxisomes in a manner similar to that induced during attack by potential fungal or oomycete pathogens [58]. Expression analysis of poplar LIMs in tension wood also suggested a connection between plant LIMs and mechanical stress [59]. Thus, rearrangement of cytoskeletal elements through actin binding and bundling may also be the mechanism of abiotic stress responses of the CRP-like *LIM* genes. Alongside, aforementioned ubiquitination mechanism of *DA1* and related proteins might also be implicated in abiotic stress resistance function of BrDAR proteins. In a very recent study, expression of *GmaDA1* gene of soybeans (*Glycine max*) showed responses to salt, drought, acid and alkali stresses and ABA treatment [60]. *AtDA1* expression occurs in response to ABA trteatment [11] and *CHS3/AtDAR4* is associated with resistance signaling and cold response in *A. thaliana*[31]. Supporting this report Bi et al. [61] hypothesized that disease resistance and environmental stress response pathways are intricately tied in plants, although the degree of overlap and the precise signaling events required for each pathway remain unclear. Thus, the results of this study indicate the likely involvement of the analyzed genes towards abiotic stress tolerance in *Brassica*.

## Conclusion

This present study identified 22 LIM domain containing genes in B. rapa and classified into two distinct functional groups. One group is plant–specific and designated as BrDAR, and another is CRP-like designated as BrLIM. These sequences were analyzed, compared them with LIM domain containing genes of other plants and analyzed their expression after applying biotic and abiotic stresses in Chinese cabbage. Overall, *BrPLIM2d, BrWLIM2a, b, c, d, e, BrWLIM1a, b, BrDAR3* and *4* responded after cold, ABA and pH stress treatments in *B. rapa*, of which *BrWLIM2c* and *BrDAR4* showed responses to *F. oxysporum* f.sp. *conglutinans* infection as well. Thus, these eight *BrLIM*s and two *BrDAR*s might be promising candidates for multiple abiotic stresses resistance, while *BrWLIM2c* and *BrDAR4* might be the most promising among them for both biotic and abiotic stresses resistance in *Brassica* crops. The remaining *BrLIM*s and *BrDAR*s might also be useful resources for induction of resistance to other biotic and abiotic stresses and/or various developmental processes of *Brassica*.

## Methods

### Plant materials

*Brassica rapa* morphotype Chinese cabbage cv. SUN-3061 plants were grown at the Department of Horticulture, Sunchon National University, Korea. Fresh roots, stems, leaves and flower buds of the Chinese cabbage plants were harvested by freezing them in liquid nitrogen and stored at -80°C to carry out subsequent organ specific expression studies.

### Stress treatments

Chinese cabbage plants were infected with *F. oxysporum* f.sp. *conglutinans* at the Screening Center for Disease Resistant Vegetable Crops, Korea. A modified version of the root-dip inoculation (RDI) method was used to inoculate the Chinese cabbage with the fungus [62]. Briefly, three week old seedlings were removed from the soil and immersed in the conidial suspension. Samples were then collected from infected and mock-infected plants at 0 h, 3 h, 6 h, 24 h, 6 d and 11 d. Upon collection, the samples were immediately frozen in liquid nitrogen, after which they were stored at -80°C until RNA isolation. The local (fourth) and systemic (fifth) leaves were harvested as samples.

For biotic stress treatments, Chinese cabbage plants were grown in soil for six weeks under culture room conditions with a 16 h light 8 h dark cycle at 25°C temperature prior to treatment. *P. carotovorum* subsp. *carotovorum* stock (10 μl) was cultured in 25 ml of liquid YEP medium until the OD_600_ was 1.4, which was equivalent to 1,170,000 colony forming units (cfu) ml^-1^, after which it was diluted to an OD_600_ of 1.19, which was equal to 1 × 10^6^ CFU ml^-1^, by adding double distilled water. For pathogen inoculation, 10 μl of *P. carotovorum* subsp. *carotovorum* culture solution (1 × 10^6^ cfu ml^-1^) was added to a freshly needle wounded site at the lower 1/3 of the midrib of the upper third leaves, after which the sample was covered with polyvinyl bags to maintain 80-90% humidity and incubated at 25°C. All inoculations were performed three times, and the infection was confirmed by observing disease lesions in the leaves of the Chinese cabbage plants. Approximately one-third of the top of the infected leaves were harvested for RNA extraction at 0 h, 6 h, 12 h, 24 h, and 72 h after inoculation, frozen in liquid nitrogen, and stored at -80°C.

For abiotic stress treatments, Chinese cabbage seeds were grown aseptically on half-strength MS (HMS) agar medium (without sucrose) with pH of 5.8 in a culture room under a 16 h light photoperiod at 25°C. After three weeks of growth, the seedlings were transferred to fresh liquid HMS medium containing 100 μM of ABA for ABA stress treatments. To induce cold stress, the seedlings were maintained at 4°C in fresh liquid HMS medium for 24 h. In addition, pH stress treatment was applied by transferring the seedlings into fresh liquid HMS medium with pH levels of 5, 7 or 9 for 24 h. For each stress, the leaf samples were collected after 0 h, 8 h and 24 h of treatment and each sample was collected three times from two plants every time. Mock treated samples (control) were collected from the seedlings after transferring into same fresh liquid HMS medium with pH levels of 5.8 during same time interval. The collected samples were frozen immediately in liquid nitrogen and stored at -80°C until RNA isolation.

### RNA extraction

Total RNA was extracted from the roots, stems, leaves, flower buds of six different stages (early to mature flower bud) and stress treated frozen samples using an RNeasy mini kit (Qiagen, USA), after which it was treated with RNase-free DNase (Promega, USA) to remove genomic DNA contaminants.

### Sequence analysis of *BrLIM*genes

We recovered 22 *LIM* genes of *B. rapa* from the *Brassica* database (http://brassicadb.org/brad/index.php) using the key word “LIM protein”. The primary structure of genes was then analyzed using protParam (http://expasy.org/tools/protparam.html). Exons were drawn using gene structure display server 2.0 (http://gsds2.cbi.pku.edu.cn/) and open reading frames (ORFs) were determined using ORF finder (http://www.ncbi.nlm.nih.gov/gorf/gorf.html). Additionally, an alignment search was conducted using NCBI BLAST (http://www.ncbi.nlm.nih.gov/BLAST/) and the BLASTp program with the nr database. Typical domains were analyzed using the EMBL web tool (http://smart.embl.de/smart/set_mode.cgi?GENOMIC=1). Multiple protein sequences were aligned using PIR (http://pir.georgetown.edu/pirwww/search/multialn.shtml) and phylogenetic tree was constructed according to the neighbor-joining method using the MEGA5.1 software (http://www.megasoftware.net) [63, 64].

### Expression analysis

#### RT-PCR analysis

AMV one step RT-PCR kit (Takara, Japan) was used for RT-PCR. Specific primers for all genes were used for RT-PCR, and actin primers of *B. rapa* (FJ969844) were used as a control (Additional file 2: Table S1). We used primer 3 program for designing primers and Tm values for forward and reverse primers of each gene was determined at 55 ± 1°C. PCR was performed using 50 ng of cDNA from the roots, leaves, stems and flower buds as templates in master mixes composed of 20 pmol of each primer, 150 μM of each dNTP, 1.2 U of *Taq* polymerase, 1× *Taq* polymerase buffer, and double-distilled H_2_O diluted to a total volume of 20 μl in 0.5 ml PCR tubes. The samples were then subjected to the following conditions: pre-denaturing at 94°C for 5 min, followed by 30 cycles of denaturation at 94°C for 30 s, annealing at 55°C for 30 s and extension at 72°C for 1 min, with a final extension for 5 min at 72°C. Microarray expression of *A. thaliana* genes were created by GENEVESTIGATOR (https://www.genevestigator.com/gv/plant.jsp).

### 3’-RACE PCR

The cDNA synthesized from *B. rapa* sample was reverse transcribed and the trapped sequence adjacent to the vector was amplified by 3’-RACE using a TAKARA 3’-Full RACE Core set according to the manufacturer’s instructions. For *BrWLIM2c* and *e*-specific PCR the primers F-1 (5’-GTG AAC ACA ACA GAG GAG GT-3’) and F-2 (5’-AGT GAG CAC AAT CCC TCT TA-3’) were used for amplification of the target portions, respectively. The PCR products were then cloned using a TOPO TA 2.1 cloning kit (Invitrogen, USA) according to the manufacturer’s instructions and sequenced with an ABI3700 sequencer (SolGent Co., Ltd., Korea).

### Real-time quantitative PCR analysis

The cDNA was synthesized using a Superscript^®^ III First-Strand synthesis kit (Invitrogen, USA) according to the manufacturer’s instructions using oligo dT primer. Real-time quantitative PCR was performed using 1 μl of cDNA in a 25 μl reaction volume employing iTaq™ SYBR^®^ Green PCR kit (Qiagen, California, USA). The same specific primers were used for real-time PCR and actin primers of *B. rapa* (FJ969844) were used as a control and the conditions for real-time PCR were as follows: 10 min at 95°C, followed by 40 cycles of 94°C for 30 s (denaturation), 58°C for 30 s (extension step), and 72°C for 45 s (signal aquisition). The fluorescence was measured following the last step of each cycle, and three replicates were used for each sample. Amplification, detection, and data analysis were conducted using a Rotor-Gene 6000 real-time rotary analyzer (Corbett Life Science, Australia). Threshold cycle (Ct) represents the number of cycles at which the fluorescence intensity was significantly higher than the background at the initial exponential phase of PCR amplification. The *Br-Actin* was used as the internal reference in all analyses and relative gene expression level was calculated on the basis of the 2^-ΔΔCt^ method [65]. Finally, the relative gene expressions in treated samples were presented as fold change compared to mock treated samples and at each time point gene expression values in mock treated samples are set to 1.

## Electronic supplementary material

Additional file 1: Figure S1: Gene structure of 22 *LIM* genes of *Brassica rapa.* Solid boxes and lines indicate exons and introns respectively. Length of exons and introns are mentioned below in base pairs (bps). **Figure S2.** Schematic representation of the existing and the postulated genomic structures of A) *BrLIM7* and B) *BrLIM9.* Solid boxes and lines indicate exons and introns respectively and their lengths are mentioned above in base pairs (bps). Specific primers were indicated by arrows, forward (→) and reverse (←), with corresponding numbers for RT-PCR expression analysis over different organs, roots (R), stems (S), leaves (L) and flower buds (Fb), and genomic DNA amplifications, which are represented beneath each genes. Primer #1 and #3, indicated by green arrows (→), were used for 3’-RACE PCR of *BrLIM7* and *BrLIM9,* respectively. (PDF 449 KB)

Additional file 2: Table S1: Primers specific for 22 *BrLIM* genes used for RT and Real time PCR analysis. **Table S2.** Sequence relatedness among 22 LIM proteins of *Brassica rapa.* (PDF 80 KB)

Additional file 3: Table S3: List of genes selected for phylogenetic analysis with sequences of deduced proteins of *B. rapa.* (PDF 164 KB)

Additional file 4: Figure S3: Microarray expressions of six *LIM*, one *DA1* and seven *DAR* genes of *Arabidopsis thaliana* after infection with *Fusarium oxysporum* (created by GENEVESTIGATOR). **Figure S4.** Microarray expressions of six *LIM*, one *DA1* and seven *DAR* genes of *Arabidopsis thaliana* after cold stress treatments (created by GENEVESTIGATOR). (XLS 44 KB)

## Abbreviations

*LIM*: Lin-11, Isl-1 and Mec-3 domains
*BrLIM*: *Brassica rapa LIM*
ABA: Abscisic acid
CRPs: Cysteine rich proteins
AFs: Actin filaments
ABPs: Actin binding proteins
kDa: Kilo Dalton
RDI: Root-dip inoculation
cfu: Colony forming units
HMS: Half-strength Murashige and Skoog
UIM: Ubiquitin interacting motif
RACE: Rapid amplification of cDNA ends.
