# Supplementary material for: Identification and characterization of LIM gene family in Brassica rapa
Source: BMC Genomics. 2014 Aug 3;15(1):641. doi: 10.1186/1471-2164-15-641 (PMC4246497; doi:10.1186/1471-2164-15-641)
Supplement: Supplementary file 1 — Additional file 1: Figure S1: Gene structure of 22 LIM genes of Brassica rapa. Solid boxes and lines indicate exons and introns respectively. Length of exons and introns are mentioned below in base pairs (bps). Figure S2. Schematic representation of the existing and the postulated genomic structures of A) BrLIM7 and B) BrLIM9. Solid boxes and lines indicate exons and introns respectively and their lengths are mentioned above in base pairs (bps). Specific primers were indicated by arrows, forward (→) and reverse (←), with corresponding numbers for RT-PCR expression analysis over different organs, roots (R), stems (S), leaves (L) and flower buds (Fb), and genomic DNA amplifications, which are represented beneath each genes. Primer #1 and #3, indicated by green arrows (→), were used for 3’-RACE PCR of BrLIM7 and BrLIM9, respectively. (PDF 449 KB) [file 12864_2014_6675_MOESM1_ESM.pdf]

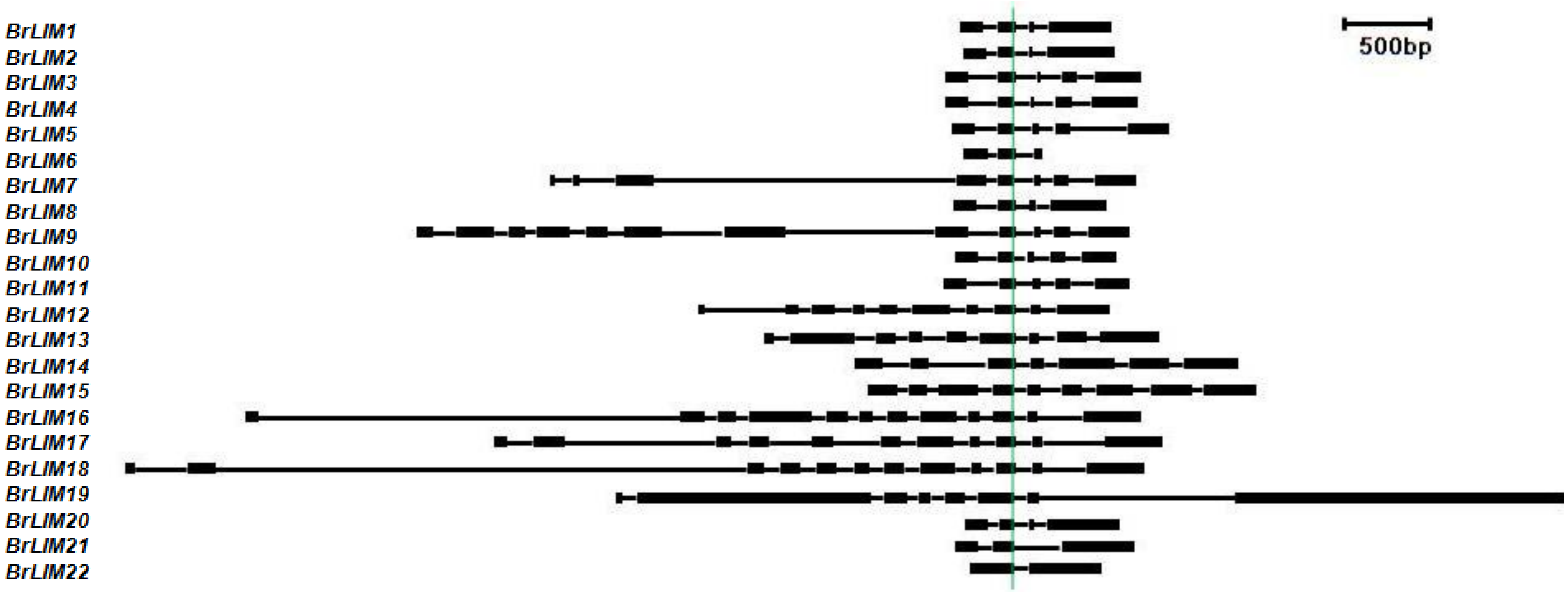

Legend: Exon intron

BrLIM1																135	90	100	78	29	84	372						
BrLIM2																135	73	100	79	26	87	393						
BrLIM3																135	176	100	128	23	121	90	99	270				
BrLIM4																135	177	100	98	23	117	90	122	270				
BrLIM5																138	129	100	106	38	93	90	335	240				
BrLIM6																138	66	100	112	41								
BrLIM7																177	66	100	112	35	82	90	157	240				
BrLIM8																135	132	100	86	38	77	327						
BrLIM9																189	186	100	103	38	81	90	110	240				
BrLIM10																135	123	97	69	44	96	90	96	207				
BrLIM11																135	194	97	103	44	89	90	135	204				
BrLIM12					36	482	74	72	133	107	75	87	99	90	213	101	63	105	119	88	59	101	302						
BrLIM13									54	102	383	116	121	77	78	135	117	80	213	78	63	94	175	87	347				
BrLIM14															156	165	103	346	164	92	84	76	333	86	232	89	317		
BrLIM15													171	72	97	80	226	90	121	75	84	119	120	83	213	106	241	69	311
BrLIM16	76	2476	137	79	106	80	367	83	121	72	84	81	120	73	213	71	63	85	119	81	59	269	332						
BrLIM17			82	148	188	887	88	106	118	251	121	280	120	93	213	91	63	92	116	105	59	364	338						
BrLIM18	61	301	170	3117	88	104	115	92	121	109	84	83	120	84	213	89	63	84	116	98	59	262	335						
BrLIM19									39	84	1373	80	130	66	75	77	123	77	213	75	66	1153	1929						
BrLIM20																	135	69	91	82	32	77	423						
BrLIM21																	135	90	129	272	426								
BrLIM22																			264	84	426								

Supplementary Fig. 1 Gene structure of 22 *LIM* genes of *Brassica rapa and a*rranged keeping their conserved regions in a vertical line. Solid boxes and lines indicate exons and introns respectively. Length of exons and introns are mentioned below in base pairs (bps)

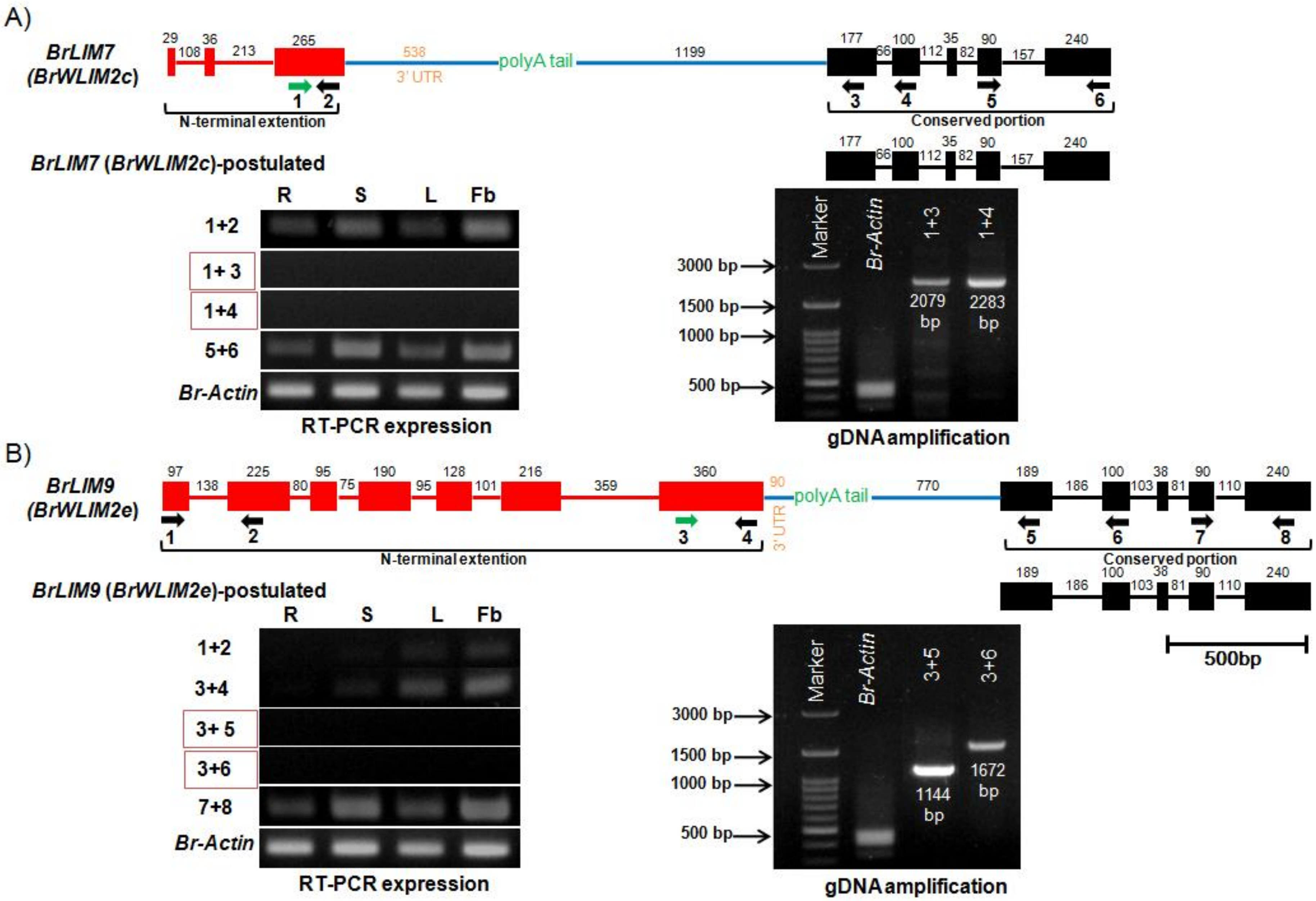

Supplementary Fig. 2 Schematic representation of the existing and the postulated genomic structures of A) *BrLIM7* and B) *BrLIM9.* Solid boxes and lines indicate exons and introns respectively and their lengths are mentioned above in base pairs (bps). Specific primers were indicated by arrows, forward (➡) and reverse (⬅), with corresponding numbers for RT-PCR expression analysis over different organs, roots (R), stems (S), leaves (L) and flower buds (Fb), and genomic DNA amplifications, which were represented beneath each genes. Primer #1 and #3, indicated by green arrows (➡), were used for 3'-RACE PCR of *BrLIM7* and *BrLIM9,* respectively.
