# Supplementary material for: Identification and characterization of LIM gene family in Brassica rapa
Source: BMC Genomics. 2014 Aug 3;15(1):641. doi: 10.1186/1471-2164-15-641 (PMC4246497; doi:10.1186/1471-2164-15-641)
Supplement: Supplementary file 2 — Additional file 2: Table S1: Primers specific for 22 BrLIM genes used for RT and Real time PCR analysis. Table S2. Sequence relatedness among 22 LIM proteins of Brassica rapa. (PDF 80 KB) [file 12864_2014_6675_MOESM2_ESM.pdf]

Supplementary Table 1. Primer information of *BrLIM* genes for RT and real-time -PCR analysis

Gene ID	Primer sequence		Product length
	Forward	Reverse	
BrLIM1	CCAAGTAAGCTATCATCCTTC	AAAGCTGACTGAAATGAACC	211
BrLIM2	GGTGGCTGCCCATTAACAC	TCATGTTCTTCGTTGGTTTCG	173
BrLIM3	CATACAAAGACTCCAAGCAAG	GAGGACGTGAGCATAGTTGC	255
BrLIM4	CATGTTTCAAGTGCGCTCAC	GCGACTTCTTCAGTGGTCTC	199
BrLIM5	CTTCTCTCAGCTGATGGTGTC	CCGGTTTAACAGGGGACTG	184
BrLIM6	CTCTCTGCTGATGGAGTTAGC	TTTATACCAGCTCAGGACTTG	211
BrLIM7	(1) GTGAACACAACAGAGGAGGT	(2) CATTGACTTTGGTGGTAGGT	180
	-	(3) AACTCCATCAGCAGAGAGAA	306
	-	(4) TTTGGTGAAGCTACCAGACT	444
	(5) CAAGAGAAATGTGCTACTTGC	(6) GATTCAGGAACCGCCGTTG	284
BrLIM8	GCCATTAACAGACAAACCAAC	CTCCGTGCGAACACTTGAAG	176
BrLIM9	(1) TTGATATCGAAGTCCCTGAC	(2) AGAGAGATTGCAGAAGCAAG	181
	(3) AGTGAGCACAATCCCTCTTA	(4) GGTGAAAATTCTTGGAGTCA	185
	-	(5) ATTTCTGCTGAGTCCCTGTA	266
	-	(6) AGGAGTTGGCTTGTCAGTTA	505
	(7) GAGCTGACAAGGACACCAAG	(8) CTCGAGCGCTGCATAGTTC	189
BrLIM10	AGATTGGGAAACCAGATAGAC	CGTGTGTGCACTTGAAACAG	177
BrLIM11	GGGACATTGTACCACAAGAG	TTATCTCCTCCTTCAAGCTG	161
BrLIM12	CGAGGTTCAAAACCATGTCTC	TACGAACTCCGTTTCTCTAGG	211
BrLIM13	AAGAAATGAGCTTGAAGCAG	CTTAAATTGTCCCTCCTCCT	248
BrLIM14	CTGGAGTGTCTAGACTCATC	TTGTTCTTCCGACAGACAG	216
BrLIM15	GATCGTGCAATCGCATTGTC	GTCCATTCCCGTAAACGTCTC	211
BrLIM16	GTTTTAGCTCACATGTGGTTG	CTGAACCCATCCCCATATGC	209
BrLIM17	CTTCCTCGTCCTCATCTTC	GAGGAGGATGATGATTTGTG	210
BrLIM18	CCTAAATGCGAAGTTTGTCAC	CACGTATTGCATGGTAAAGTG	262
BrLIM19	AGCTTAGGTAAAGACGCTGAC	AACAAAGGCTTCGACCAGAAC	210
BrLIM20	GAATGTTTCCACAAGACTTGC	CAGGTTTGTGGTTGTCATGAG	220
BrLIM21	ACATGGAAGGTGAATGTTACC	GATCATCAGAAGGCGGAGTAG	222
BrLIM22	TCTCTCAACGGTGTACTCTAC	TTCAGGAGCTGCTTCTTCTTC	210
Br-Actin (Real-time)	CAACCAATCGTCTGTGACAA	ATGTCTTGGCCTACCAACAA	105
Br-Actin (RT-PCR)	ATGGCCGAGGCTGATGACAT	CGGTTCTTCTTACCGAGGCT	330

Supplementary Table 2. Sequence relatedness among the 22 LIM proteins of *Brassica rapa**

	a	b	c	d	e	f	g	h	i	j	k	l	m	n	o	p	q	r	s	t	u	v
BrLIM1 (a)	100																					
BrLIM2 (b)	87	100																				
BrLIM3 (c)	76	77	100																			
BrLIM4 (d)	74	76	95	100																		
BrLIM5 (e)	63	60	58	59	100																	
BrLIM6 (f)	65	65	63	61	96	100																
BrLIM7 (g)	62	62	59	60	97	99	100															
BrLIM8 (h)	63	63	62	63	90	82	91	100														
BrLIM9 (i)	63	62	62	63	86	83	87	97	100													
BrLIM10 (j)	51	51	51	51	57	53	57	58	58	100												
BrLIM11 (k)	49	51	51	51	58	53	58	59	59	98	100											
BrLIM12 (l)	80	80	67	67	60	57	60	60	57	63	63	100										
BrLIM13 (m)	37	37	60	37	33	57	33	37	37	46	43	55	100									
BrLIM14 (n)	29	35	30	26	29	24	29	29	29	32	32	48	48	100								
BrLIM15 (o)	29	29	80	80	56	24	29	29	71	57	57	63	48	93	100							
BrLIM16 (p)	37	39	80	46	37	37	37	40	86	40	40	51	52	62	61	100						
BrLIM17 (q)	43	43	36	36	28	23	24	24	43	29	29	57	43	78	52	53	100					
BrLIM18 (r)	27	25	38	38	27	38	31	33	30	44	44	47	45	49	60	59	85	100				
BrLIM19 (s)	57	75	35	42	75	75	75	50	29	63	63	69	42	39	67	52	40	39	100			
BrLIM20 (t)	74	82	71	71	58	66	58	61	60	48	49	83	46	40	29	39	50	33	38	100		
BrLIM21 (u)	77	80	74	77	60	61	59	61	58	48	49	83	57	25	28	44	43	33	57	82	100	
BrLIM22 (v)	87	88	77	78	62	60	61	63	59	50	51	83	41	25	28	39	43	33	75	83	92	100

*Results from pairwise amino acid sequence comparisons are shown as percent identity among members of the LIM family proteins.
