# Supplementary material for: Identification and characterization of LIM gene family in Brassica rapa
Source: BMC Genomics. 2014 Aug 3;15(1):641. doi: 10.1186/1471-2164-15-641 (PMC4246497; doi:10.1186/1471-2164-15-641)
Supplement: Supplementary file 3 — Additional file 3: Table S3: List of genes selected for phylogenetic analysis with sequences of deduced proteins of B. rapa. (PDF 164 KB) [file 12864_2014_6675_MOESM3_ESM.pdf]

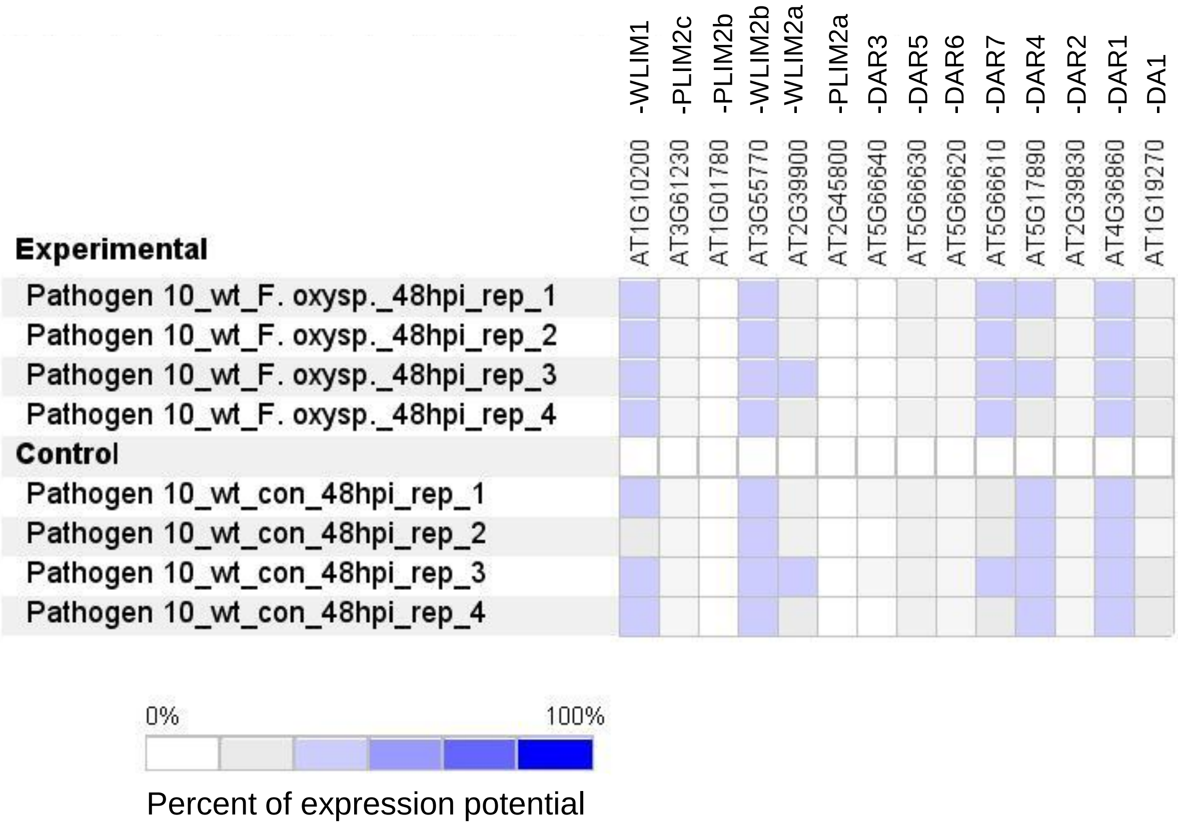

Supplementary Fig. 3 Microarray expressions of six *LIM*, one *DA1* and seven *DAR* genes of *Arabidopsis thaliana* after infection with *Fusarium oxysporum* (created by GENEVESTIGATOR)

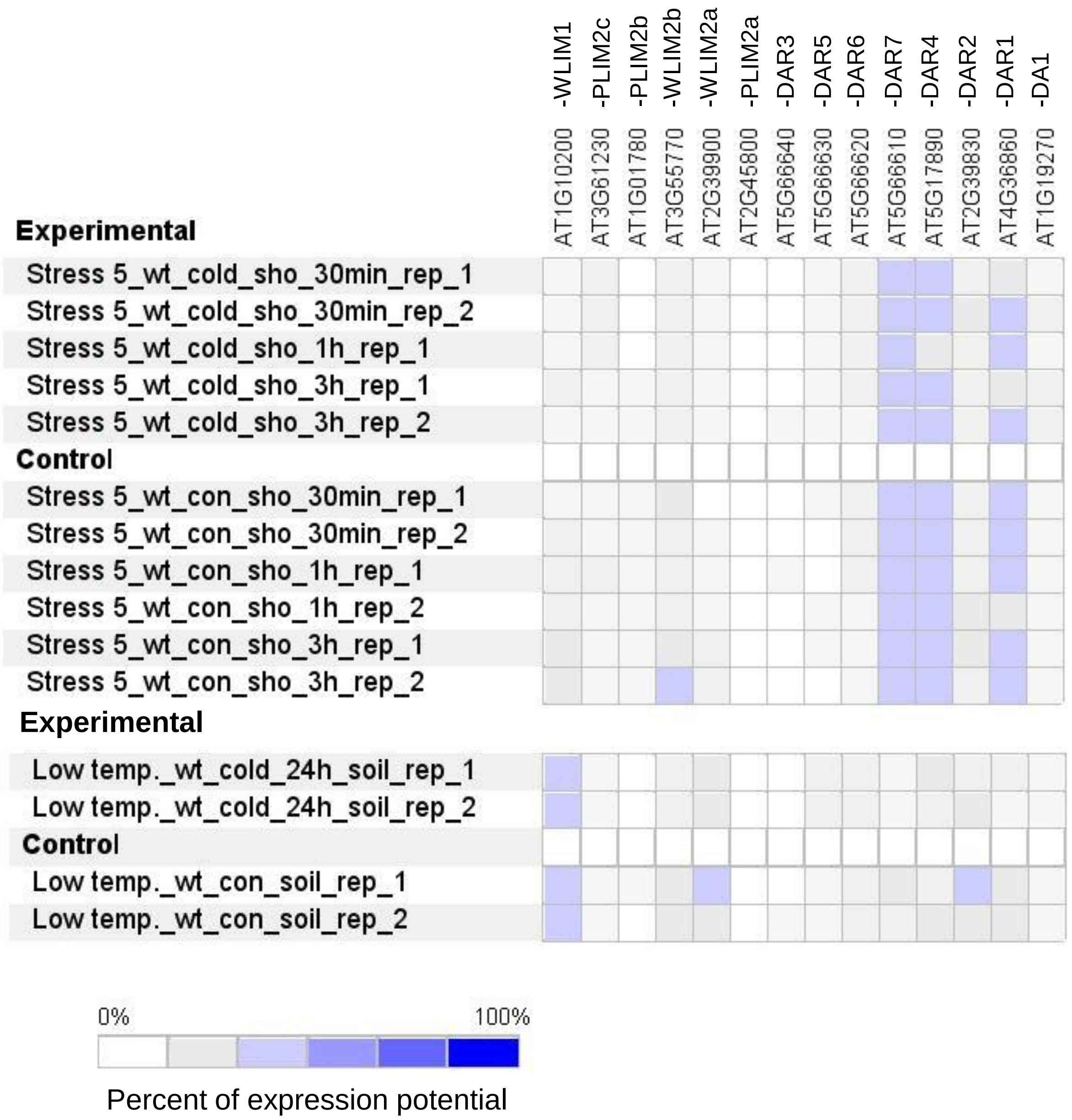

Supplementary Fig. 4 Microarray expressions of six *LIM*, one *DA1* and seven *DAR* genes of *Arabidopsis thaliana* after cold stress treatments (created by GENEVESTIGATOR)
